# Tweens’ Wishful Identification and Parasocial Relationships With YouTubers

**DOI:** 10.3389/fpsyg.2019.02781

**Published:** 2019-12-17

**Authors:** Amanda N. Tolbert, Kristin L. Drogos

**Affiliations:** ^1^Department of Communication, University of Illinois at Urbana–Champaign, Champaign, IL, United States; ^2^Research Center for Group Dynamics, University of Michigan, Institute for Social Research, Ann Arbor, MI, United States

**Keywords:** YouTube, gender identity, tween, parasocial relationship, wishful identification

## Abstract

Children between the ages of 9 and 12 – commonly called tweens – are one of the fastest growing audiences for YouTube content. The current study explores how tweens are watching YouTube and the nature of their parasocial relationships and wishful identification with their favorite YouTube personalities. Results show that tweens identified gender-congruent YouTubers as their favorite. Moreover, tweens perceived male and female YouTubers to have different attributes. For instance, male YouTubers were rated as more violent than female YouTubers, and female YouTubers were rated as more attractive and popular than male YouTubers. Gender also played a role in attachment patterns. Tween boys’ wishful identification was predicted by YouTubers who were violent and funny and their parasocial relationships were predicted by YouTubers who were funny, successful, and attractive. Meanwhile, tween girls’ wishful identification was predicted by YouTubers’ who were funny, and their parasocial relationships were predicted by YouTubers’ who were funny and popular. Results are discussed in terms of gender socialization theory.

## Introduction

YouTube has over 2 billion users per month (Spangler, 2019), and a billion hours of video are watched daily (YouTube for Press, n.d.). Tweens, children between nine and 12 years old, are one of the fastest growing groups of YouTube viewers. The most recent nationally representative research found that more than twice as many United States tweens watch online videos every day than did in 2015, increasing from 24% daily viewers to 56% (Rideout and Robb, 2019). This same report found that watching online videos is tweens’ favorite media activity. A similar report from the United Kingdom shows that half (49%) of tweens prefer to watch YouTube content over television programing (OFCOM, 2019).

YouTube personalities, or “YouTubers,” have emerged as a new kind of celebrity, accumulating millions of fans and earning tens of millions of dollars annually. Compared to traditional media wherein tweens merely view content, YouTube allows tweens to interact with their favorite YouTuber by commenting on videos and sometimes directly communicating with the YouTuber. Tweens can also engage in various ways when they are not using the YouTube platform. For instance, they can purchase YouTubers’ merchandise, see them at sold-out arenas, watch them on television events such as the Nickelodeon Kids’ Choice Awards, or follow them across other social media platforms. With ample opportunity to engage with YouTubers, it stands to reason that tweens are forming attachments to these new celebrities.

Previous research on children’s character attachments has focused on the psychological mechanisms of wishful identification (WI) and parasocial relationships (PSR) with mass media characters. This body of work reveals that (1) children can form these attachments; (2) there are gender differences in how attachments are formed; and (3) WI and PSR function as mediators between media exposure and effects. Given the differing levels of interactivity between mass media and YouTube, new patterns of WI and PSR may be forming between children and their favorite YouTuber. The purpose of this study is to understand more about tweens’ use of YouTube and the nature of WI and PSRs that tweens are forming with their favorite YouTubers.

## Literature Review

### Tween Use of YouTube

Research on tweens’ viewing relationships with YouTubers is beginning to emerge. One study found that 40% of tweens reported watching YouTube specifically to view YouTubers (Children and parents: Media use and attitudes report 2018, 2019). In a survey of United States tweens, 34% could name a favorite YouTube show, channel, or star (Rideout, 2015). When tweens are specifically asked about their *favorite* YouTubers, Folkvord et al. (2019) found that more than 40% of tweens reported that, on days when they watch their favorite YouTuber, they generally watch for more than 1 h. Additionally, 24% of tweens reported watching their favorite YouTuber every day of the week.

One explanation for the popularity of YouTubers is that the time tweens are spending on YouTube is increasing. Recent data shows a significant increase in the amount of time American tweens spend watching YouTube, jumping from 25 min a day in 2015, to 56 min a day in 2019 (Rideout and Robb, 2019). Data from the United Kingdom indicates that this trend extends outside of the United States. In 2018, a majority (77%) of tweens in the United Kingdom reported using YouTube (Children and parents: Media use and attitudes report 2018, 2019). These data confirm that YouTube has become an increasing presence in the lives of tweens across the globe.

Another reason tween viewership has seen an increase is due to ease of access. Over half (52%) of American tweens have their own tablet, 41% have their own smart phone, and 23% have their own laptop computer (Rideout and Robb, 2019). Similar statistics can be found in the United Kingdom, where 47% of tweens own their own tablet and 35% have their own smartphone (Children and parents: Media use and attitudes report 2018, 2019). These personal devices are often used to watch digital videos. YouTube reports that 70% of their watch time comes from mobile devices (YouTube for Press, n.d.). More specifically tweens’ YouTube viewing is primarily done on smartphones and tablets (Rideout and Robb, 2019).

Although data have indicated that YouTube is extremely popular among tweens, when compared to what we know about youth television viewing habits, we know very little about the specific contexts of tween’s YouTube viewing. For example, we do not what time of day they are watching, or if they watch more on weekdays than on weekends. We also do not know if tweens typically co-view with friends or family or watch alone. Over half (61%) of American parents with children under eleven reported that their child has encountered YouTube content that was “not suitable for children” (Smith et al., 2018). One way to mitigate the potential negative effects of consuming inappropriate content is through parental co-viewing (Nathanson, 1999). Yet we do not know if co-viewing YouTube is prevalent. One purpose of the present study is to begin filling these gaps by exploring how and with whom tweens are viewing YouTube. Given how little is known, we propose the following question:

RQ_1_: In what contexts do tweens watch YouTube?

Evidence has begun to emerge that tweens are absorbing information and learning new ideas and behaviors from YouTube videos. Martínez and Olsson (2019) found that tweens use YouTubers as a source of information and informal learning. There are also reports of children as young as 5 pretending to have their own YouTube channel, narrating their lives and playtime as if they were speaking into a camera, always reminding their pretend viewers to “subscribe” to their channel (Pierson, 2016). Social Learning Theory posits that new behaviors are learned through the observation and imitation of others (Bandura, 1977). More specifically, models that get children’s attention, are highly attractive, and most similar to the self are most likely to produce social learning effects (Bandura, 2001). Therefore, YouTubers who are attractive and popular, and considered to be like the self should be potent role models for young age groups.

One way in which tweens may consider themselves similar to YouTubers is through gender identity. In line with social cognitive theory, gender schema theory posits that children watch members of their own gender to learn how to behave as a male or female (Bem, 1981). More specifically, gender schema theory suggests that once children know their gender, they begin forming mental conceptions of activities, norms, attributes, and scripts that are associated with being male or female. These mental conceptions, or schemas, influence how children encode and interpret gender-related information (Leaper, 2015). Tweens are at an interesting developmental stage when it comes to gender identity development. As they advance out of rigid, essentialist thinking associated with early childhood, they begin to adopt more flexible gender attitudes (Halim and Ruble, 2010). Any agent that influences gender socialization during the tween years has the potential to shape the development and flexibility of gender schemas.

Previous research has established that child gender plays a role in attachments to television and film characters. In one early study, Reeves and Greenberg (1977) presented a list of popular characters to children ages 8, 10, and 12, and had them rate the characters on various dimensions. Children from all three age groups rated same-sex characters more positively than opposite-sex ones. Similarly, Hoffner (1996) asked 155 s through sixth graders to name their favorite TV character. She found substantial differences in children’s choices. Nearly all the boys chose a male character, whereas roughly half the girls named a female character. Other research has found similar patterns among children as early as preschool: almost all of the preschool boys in one sample chose a male character as their favorite, as did a majority (61%) of girls (Wilson and Drogos, 2007). In a study of 370 middle schoolers, Steinke et al. (2006) found that boys identified more with a male scientist character, while girls identified more with female scientist characters.

Past research has focused on children’s attachments to media characters featured in traditional mass media outlets such as television and film. To date no one has studied how children form attachments to interactive media celebrities like YouTubers. There is reason to speculate that there may be differences between tweens’ favorite mass media characters and their favorite YouTubers. For instance, Rosaen and Dibble (2008) found that social realism, defined as the perception that a character could exist in the real world, is positively related to character attachments. Many traditional media characters are quite fantastical and cannot be considered socially real (e.g., superheroes, anthropomorphized cartoons), while most YouTubers can, and do, exist in the real world. In fact, some popular YouTubers began their careers as “normal” people producing videos from their homes before they achieved celebrity status. Furthermore, it stands to reason that social realism may increase through the commenting and private messaging affordances of the platform.

Another difference that may impact tweens’ choices in favorite characters is that there remains an overrepresentation of male characters on television and film (see Smith and Granados, 2009), which may be why most boys choose male characters as their favorite, whereas girls may or may not choose a female character as their favorite. Although it is not certain if there are more male YouTubers than female YouTubers, there are certainly more opportunities to watch female YouTubers. Ethnographic evidence suggests that YouTubers can play a role in tweens’ construction of gender identity (Martínez and Olsson, 2019), and gender schema theory would predict that tween girls may selectively seek out female YouTubers and form attachments to them over male YouTubers, whereas tween boys will be likely to seek out and prefer male YouTubers to female YouTubers. It is with this rationale that we pose our first hypothesis:

H_1_: Tweens will select gender-congruent YouTubers as their favorite more often than they will select YouTubers of a different gender.

Research has also found that boys and girls perceive different qualities in their favorite male and female characters. For instance, preschool girls were found to identify more with characters who were sassy whereas boys’ identified based on the strength of the character (Wilson and Drogos, 2007). Hoffner (1996) found that both tween boys and girls rated favorite female characters as more prosocial in their behavior than they did favorite male characters. In addition, girls rated favorite female characters as more physically attractive and more intelligent than they did favorite male characters. Mirroring this research, we anticipate differences in how viewers experience male and female YouTubers. Thus, we ask:

RQ_2_: How does gender of the YouTuber influence tweens’ perceptions of their attributes?

### Wishful Identification

As tweens seek out content and merchandise produced by their favorite YouTuber, their attraction may move into an emotional connection that exists outside of their mediated experience. One manifestation of this type of connection is *wishful identification.* Wishful identification (WI) is the psychological desire to be like a media personality (Feilitzen and Linne, 1975). This desire can lead to sharing a similar perspective with the character (Hoffner, 1996). WI moves beyond merely liking a character – it is the psychological attachment between a viewer and a character that leads to imagining the self as being the character (Cohen, 2001). In other words, WI signals aspiring to emulate a character. Given that being a YouTuber was the most desirable profession among a global sample of tweens, there is reason to speculate that tweens want to imitate YouTubers (LEGO Group, 2019).

Research shows that WI is a moderator of the psychological and social effects of the media on young people. Bond and Drogos (2014) found that young adults who identified with personalities from *Jersey Shore*, a risqué reality television series, were more likely to have permissive sexual attitudes compared to those who did not identify with the reality TV personalities. Research by Harrison (1997) found that wishing to be like a thin media personality is a significant predictor of eating disorders. Similarly, other research shows that identifying with violent video game characters is related to players’ real-life aggression (Konijn et al., 2007). In a more positive vein, research has shown that viewers also identify with characters who portray prosocial behaviors (Ramasubramanian and Kornfield, 2012). Additionally, Ward (2004) found that identification with popular Black characters was associated with higher self-esteem among Black high schoolers.

It stands to reason that WI will be related to how much time tweens spend watching YouTube. Tweens who seldom watch will be less familiar with the content produced by the masses of YouTube personalities. Increased time spent watching television has been correlated to increases in WI among children (Hoffner, 1996). Based on this reasoning, we predict:

H_2_: Wishful identification among tweens will be positively related to time spent on YouTube.

Several studies have attempted to identify the character traits that are linked with the experience of WI with media characters. For instance, WI with a mass media character is stronger for children if the character and child are of the same gender (Lonial and Van Auken, 1986). In fact, overall perceived similarity with media characters tends to lead to higher rates of WI (Hoffner and Buchanan, 2005). We predict that tweens’ WI will follow similar patterns:

H_3_: Tweens’ wishful identification with YouTubers will be positively related to perceived similarity to self.

Additionally, Hoffner and Buchanan (2005) found that WI is related to gender-based character attributes. More specifically, young adult males identified more with male characters who were perceived as being violent, successful, and intelligent, while females identified with female characters who were perceived as being successful, attractive, intelligent, and popular. This begs the question of how these gender patterns will influence WI for tween boys and girls:

RQ_3a_: Which perceived YouTuber characteristics will be related to wishful identification for tween boys?

RQ_3b_: Which perceived YouTuber characteristics will be related to wishful identification for tween girls?

### Parasocial Relationships

Another form of attachment that tweens may be forming with YouTubers is the parasocial relationship. A parasocial relationship (PSR) is conceptually related to but distinct from WI. Parasocial interaction involves the emotions, thoughts, and actions that a viewer experiences during media exposure that are geared toward a specific performer or character (Cohen, 2009). When experienced repeatedly, over time these interactions can develop into a PSR, which is a one-sided symbolic relationship between the viewer and a media character. That is to say, a PSR refers to the feeling of friendship that a viewer develops toward a media character. In their foundational conceptualization of PSRs, Horton and Wohl (1956) described this relationship with characters as a sense of “intimacy at a distance” experienced by the viewer.

PSRs are a normal occurrence in traditional media environments and are experienced both by adults and by children (Hoffner, 1996; Rosaen and Dibble, 2008). Much like WI, PSRs play an important role in the moderating effects of media consumption. For instance, PSRs have been related to children’s learning outcomes. Howard Gola et al. (2013) found that children are more likely to learn an academic lesson that comes from a character they find familiar compared to a character they do not know. Additionally, to learn from a character, children need to feel that the character resembles a person (Bond and Calvert, 2014). Considering the majority of celebrity YouTubers are real people, and tweens are watching enough to become familiar with their favorite YouTubers, it stands to reason that tweens are likely forming PSRs and learning from these YouTube personalities.

Researchers have recently started to explore PSRs in new media environments, such as *via* social media platforms. Kim and Song (2016) found that celebrity self-disclosure, and the resulting perception of social presence, positively affected parasocial interaction on Twitter. Additionally, PSRs on social media have been linked to changes in attitude and behavior. For instance, researchers recently found that PSRs were related to perceived source-trustworthiness, which has a positive effect on the perception of brand credibility and leads to purchase intention (Chung and Cho, 2017). However, PSR research in the realm of emerging media is quite nascent. To date there have been no studies on children’s PSRs with new media personalities. We do know that children’s PSRs with mass media characters develop through repeated exposure over time, similar to real-life friendship development (Calvert and Richards, 2014). Recent research also suggests that when tweens feel a strong bond with their favorite YouTuber, they will spend more time viewing the YouTuber’s content (Folkvord et al., 2019). As such, we predict a similar time-related pattern on YouTube:

H_4_: Tweens’ parasocial relationships will be positively related to time spent on YouTube.

In children, PSRs have been positively related to the social realism of television characters (Rosaen and Dibble, 2008). A human YouTuber would be considered more socially real than the many television characters who are cartoons, depicted as magical, or are otherwise unrealistic. Furthermore, Turner (1993) found that individuals are more likely to form PSRs with media characters whom they perceive as similar to themselves. We hypothesize that similarity to self will also play a role here:

H_5_: Tweens’ parasocial relationships with YouTubers will be positively related to perceived similarity to self.

Finally, results from a handful of studies suggest that different character attributes predict PSRs for boys and girls. Hoffner (1996) found that intelligence and strength predicted boys’ PSRs, while attractiveness predicted girls’ PSRs. Bond and Calvert (2014) found that as young children grow older and transition to new favorite media characters, girls’ new favorites tend to be more feminine, while boys’ become more masculine. Given the paucity of research on children’s PSRs with new media personalities, we pose the following questions:

RQ_4a_: How do the perceived characteristics of YouTubers relate to tween boys’ parasocial relationships with their favorite YouTuber?

RQ_4b_: How do the perceived characteristics of YouTubers relate to tween girls’ parasocial relationships with their favorite YouTuber?

Although YouTube viewership can be similar to mass media viewership, there are distinct differences in how the platform is used, which can alter the viewing experience for audience members. For instance, YouTube viewers can interact with content creators in comment sections on the platform as well as across other social media platforms. In fact, it is not uncommon that a YouTuber will participate in the conversations in his or her comments section. Additionally, YouTube has direct messaging abilities that allow viewers and content creators to privately interact with one another. Finally, not only do YouTubers and viewers interact with one another, but viewers also interact with other viewers, fostering a palpable sense of community on a YouTuber’s page (Rotman and Preece, 2010). Given these differences, it seems plausible that YouTube fosters different types of attachment patterns between children and their favorite YouTuber compared to those built with traditional media characters. Giles (2002) argues that representation of a media character across different media outlets should foster parasocial interaction. It may be that “spending more time” with a favorite YouTuber across several social media platforms strengthens PSRs. However, content that is placed on YouTube is often replicated across other social media sites, so following a YouTuber across many social media platforms may not breed the feelings of intimacy needed to strengthen a PSR. Given that these two options are possible, we pose the following question:

RQ_5_: Are parasocial relationships related to following a YouTuber on other social media platforms?

Compared to traditional mass media, one of the affordances of YouTube is the ability to interact with the content producer. A viewer can leave comments on a YouTuber’s videos and send private messages to a YouTuber. It is not uncommon to receive a response from a celebrity on social media (Marwick and Boyd, 2011). Conceivably, receiving a response from a YouTuber could increase the feeling that the YouTuber is a friend. Thus, we pose the following hypothesis:

H_6_: Parasocial relationships among tweens will be positively related to receiving responses from the YouTuber.

## Materials and Methods

### Participants

A total of 161 children, aged 9 through 12 years old, participated in the study. Participants were recruited in a large, metropolitan area in the southern United States through local school districts, local public libraries, and events at a university. Participants received a $5 debit gift card. The sample (*N* = 161) was 52% female and 48% male, with an average age of 10.78 years (*SD* = 0.822). Over half (59%) of participants indicated that they were white, 23% were of mixed race, 6% were Asian, 5% were Hispanic or Latino, 4% were Black, and 3% of the participants marked “Other” as their race.

### Procedure

After parental consent and child assent were obtained, researchers administered a paper-and-pencil survey to mid-sized groups (10–20) of children at a time. A researcher read each survey item aloud as participants marked their answers.

### Measures

#### Time on YouTube

In order to understand the nature of YouTube use, participants were asked about the time they spend on YouTube. Participants reported how much time was spent on YouTube during different parts of an average weekday and an average week*end* day. An example item from the scale asked, “On the average weekday how much total time do you spend on YouTube after school but before dinner?” Response options included, 0 (No time), 1 (1–30 min), 2 (31–60 min), 3 (1–11/2 h), 4 (11/2–2 h), 5 (2–21/2 h), 6 (21/2–3 h), and 7 (3+ h). Each participant received a score indicating his or her average time spent on YouTube during the weekday (*M* = 5.41, *SD* = 4.97), weekend days (*M* = 9.88, *SD* = 8.03), and full week (*M* = 15.34, *SD* = 12.21). Higher scores indicated more time spent.

#### Device Use

Participants were asked which device(s) they use to watch YouTube and were directed to mark all answers that apply. Answer options included (1) Laptop/Computer, (2) Cell phone, (3) Tablet, (4) Gaming console, (5) Other.

#### Social Viewing

Three items were used to assess how often tweens watch YouTube with other people. Participants were asked to identify how often they watched YouTube (1) alone, (2) with their parents, and (3) with their friends. Participants responded using a 5-point Likert-type scale ranging from 1 (never) to 5 (every time).

#### Favorite YouTuber

Participants identified their favorite YouTube personality by writing his or her name and/or the YouTube channel name. Participants also provided the gender, approximate age, and racial demographics of the YouTuber. It should be noted that once participants identified their favorite YouTuber they were asked to write the name of the YouTuber on each of the remaining pages of the survey. Subsequently every time participants moved to a new page in the survey, the researchers could remind them to review their favorite YouTuber and answer the questions accordingly.

#### Parasocial Relationships

A 5-item scale was used to measure the intensity of PSRs (α = 0.73; Eyal and Cohen, 2006) with YouTubers. Participants were asked to think of the previously identified YouTube personality as they completed the items on the scale. An example item from the scale includes, “I like my favorite YouTuber.” Participants responded using a 5-point Likert scale ranging from 1 (strongly disagree) to 5 (strongly agree). Responses were averaged (*M* = 3.83, *SD* = 0.81). Higher scores indicated a stronger PSR with the YouTuber.

#### Wishful Identification

A 5-item scale was used to measure wishful identification (α = 0.77; Hoffner and Buchanan, 2005). Participants were asked to think of their favorite YouTuber as they completed the items on the scale. An example item from the scale included, “I wish I could be more like my favorite YouTuber.” Participants responded using a 5-point Likert scale ranging from 1 (strongly disagree) to 5 (strongly agree). Average scores were computed for each participant where higher scores represented a stronger sense of WI with their favorite YouTube personality (*M* = 3.56, *SD* = 0.90).

#### Social Media Following of YouTuber

To test if cross-platform exposure influenced attachment to their favorite YouTubers, participants were asked to indicate whether they followed their favorite YouTuber on Instagram, Facebook, Twitter, Snapchat and Musical.ly (now known as TikTok). A sum score was computed, ranging from 0 to 4, with higher scores indicating following the YouTuber on more social media platforms (*M* = 0.79, *SD* = 1.11).

#### Interpersonal Engagement With YouTuber

Participants answered questions about the frequency that they received communications from a YouTuber. One item assessed the number of times a YouTuber responded to private messages sent by the participant, and another item measured the number of times a YouTuber responded to a comment left by the participant. Responses were recorded on a 6-point scale ranging from “0 times” to “5 times or more.”

#### Perceived Attributes

To measure the perceived character attributes of the chosen YouTube personality, a set of six scales were adapted from Hoffner and Buchanan (2005). Participants were asked to indicate the extent to which they agreed that several items described their favorite YouTube personality, using a 5-point Likert scale that ranged from 1 (strongly disagree) to 5 (strongly agree). The items were presented in a random order and measured the following six attributes as defined by Hoffner and Buchanan: *smart* (smart, intelligent, stupid [reversed]; α = 0.88), *successful* (successful, achieves goals; α = 0.64), *attractive* (physically attractive, ugly [reversed], good-looking; α = 0.71), *funny* (funny, humorous, makes me laugh; α = 0.83), *popular* (respected by others, receives approval, has lots of friends, well liked, gets support from others; α = 0.72), and *violent* (does violent things, physically hurts people; α = 0.67).

#### Perceived Similarity

Perceived similarity to the participant’s favorite YouTube personality was measured using the attitude similarity subscale of the Perceived Homophily Measure (α = 0.66; McCroskey et al., 1975). Participants reported on a semantic differential scale with options ranging from 1 to 7. Scores could range from 4 to 28, with higher scores indicating greater perceived similarity (*M* = 16.62, *SD* = 4.84). For instance, items included the stem “My favorite YouTuber…” (1) “doesn’t think like me” to (7) “thinks like me” and (1) “doesn’t behave like me” to (7) “behaves like me.”

## Results

### YouTube Viewing Contexts

The first research question asked about the patterns of tweens’ YouTube use. Almost all (98%) of the tweens in this study reported watching YouTube. Only three participants reported not watching YouTube. Those who did watched on a variety of devices. Almost three-quarters (73%) of the sample accessed YouTube on a tablet. Watching on a cell phone was almost as popular, with 70% of the sample reporting doing so. And a majority of tweens (65%) reported watching on a laptop or computer. It was less common to watch YouTube through a videogame console, but even so, almost one-third (28%) of the sample reported watching YouTube this way.

In order to assess how much time was spent on YouTube each week, tweens were asked two sets of questions about time spent on the average weekday and on the average weekend day. On average, tweens spent about 15–30 min watching YouTube every day throughout the week. They reported spending significantly more time watching YouTube on the average weekend day (*M* = 2.09, *SD* = 1.66) than on the average weekday (*M* = 1.12, *SD* = 1.09; *p* < 0.001). The most popular time to watch YouTube was in the evening “after dinner but before bed” with a majority (74.5%) of tweens reporting spending some time on YouTube during this time period both during the week and on the weekends. Interestingly, when asked if they watch YouTube “after bed when they are supposed to be asleep,” roughly two out of five tweens admitted to spending at least some time watching YouTube when they were supposed to be sleeping during the weeknights (36.3%), and weekend nights (40.8%).

Participants also indicated with whom they watched YouTube content. About half (52%) of the participants reported watching YouTube by themselves “almost every time” and 21% reported watching alone “every time” they watched. It was also common to watch with friends, with three out of five participants (58%) in the sample reporting doing so “sometimes.” It was extremely rare for participants to report watching YouTube with parents. In fact, a majority (82%) of tweens in this sample reported co-viewing YouTube with their parents “never” or “almost never.” Only 2 individuals (1%) reported watching YouTube with a parent “every time.”

### Favorite YouTubers

An overwhelming majority of participants (91.9%) reported having a favorite YouTuber. Only 13 out of 161 participants said they did not have a favorite YouTuber, and thus were excluded from further analysis. Participants identified a total of 98 unique YouTubers as their favorite, demonstrating the vast array of YouTubers who attract tweens. The most popular YouTubers listed were DanTDM (3.7%), Ali-A (3.1%), and The Odd 1s Out (3.1%). A full list of all YouTubers reported is available in Appendix A.

There was little diversity in terms of the age and race of the YouTubers. Participants identified a majority (87%) of the YouTubers as over the age of 18, with only 6% listed as 10 to 12-years-old, 5% between 13 and 15-years old, and only 1% between 16 and 18-years old. A majority (65%) of YouTubers listed were identified as white, 10% were identified as mixed race, 6% as Black or African American, 4% as Asian, 3% as Hispanic or LatinX, and 2% were identified as “other.”

In total 96 male and 51 female YouTubers were chosen as favorites. The first hypothesis proposed that male participants would be more likely to choose male YouTubers, and female participants more likely to choose female YouTubers. A chi-square analysis on the frequency of male and female YouTubers by gender of the participants was significant, χ^2^(1,*N* = 147) = 63.44, *p* < 0.001. In support of Hypothesis 1, almost all tween boys (97.2%) selected a same-gender YouTuber as their favorite and a majority of tween girls (65.3%) selected a female YouTuber as their favorite (see Table 1). Although girls were more likely to report a female YouTuber as their favorite, a little over one-third of the sample (34.7%) reported a male as their favorite. Even so, a *post hoc* showed that girls were significantly more likely to report a female YouTuber than a male YouTuber (*M* = *0.65*; *SD* = *0.48*); *t*(74 = 11.81, *p* < 0.001).

**TABLE 1:** Results of chi-square test and descriptive statistics for gender congruency between YouTuber and child.

	**Child Gender**	
**YouTuber Gender**	**Boy**	**Girl**
Male	70 (97.2%)	2 (2.8%)
Female	26 (34.7%)	49 (65.3%)

*χ^2^ = 63.45, *p* < 0.001, df = 1. Numbers in parentheses indicate column percentages.*

The second research question asked how tweens perceived different characteristics between male and female YouTubers. Independent-samples *t*-tests were conducted to test the differences. Female YouTubers were rated as more attractive (*M* = 4.01; *SD* = 0.77) than male YouTubers (*M* = 3.42; *SD* = *0.85*); *t*(139 = −4.02, *p* < 0.001) and as more popular (*M* = 4.42; *SD* = *0.57*) compared to male YouTubers (*M* = 4.18; *SD* = 0.71); [*t*(138) = −1.98, *p* = 0.05]. Female YouTubers tended to be perceived as more successful (*M* = 4.56, *SD* = 0.55) compared to male YouTubers (*M* = 4.31, *SD* = 0.85); [*t*(145) = −1.87, *p* = 0.06]. Meanwhile, male YouTubers were rated as more violent (*M* = 1.39*; SD* = 0.84) than their female counterparts (*M* = 1.13; *SD* = *0.53*); [*t*(140) = 1.99, *p* < 0.05]. There were no differences between males and females on their rated intelligence, or humor.

### Tweens Wishful Identification With YouTubers

Tweens could score from 1 to 5 on the WI index. The mean score was 3.6 (*SD* = 0.90), indicating that tweens did experience WI with their favorite YouTuber. In all, half of the participants had scores at or above the mean, and about one-third (30%) of participants had WI scores at or above 4, signaling that many tweens had strong WI with their favorite YouTuber.

The second hypothesis proposed that time spent on YouTube would be positively related to WI. A hierarchical linear regression was conducted in which tweens’ demographic variables (age, race, and sex) were entered into Block 1. These variables did not predict WI. Time spent on YouTube was entered into Block 2. This block was significant (β = 0.26, *p* < 0.01) such that more time spent on YouTube was related to higher WI scores (see Table 2). Thus, H2 was supported.

**TABLE 2:** Summary of hierarchical regression for time spent on YouTube predicting wishful identification.

	**B**	***SE***	**β**	**ΔR^2^**
**Block 1**				
Race	0	0.04	0.01	
Age	–0.03	0.09	–0.02	
Gender	–0.10	0.15	–0.05	0
**Block 2**				
Time Spent on YouTube	0.20	0.06	0.26^∗∗^	0.07^∗^

*All coefficients are from the full model. ^∗^*p* < 0.05, ^∗∗^*p* < 0.01.*

The third hypothesis predicted that perceived similarity between the participant and YouTuber would be positively related to tweens’ WI. A hierarchical linear regression was run in which participant demographics and time spent on YouTube were controlled in Blocks 1 and 2, respectively (see Table 3). Block 3 included perceived similarity, which was positively related to WI (β = 0.26, *p* < 0.01). Thus, H3 was supported.

**TABLE 3:** Summary of hierarchical regression for perceived similarity predicting wishful identification.

	**B**	***SE***	**β**	**Δ*R*^2^**
**Block 1**				
Race	0.05	0.03	0.10	
Age	–0.07	0.09	–0.06	
Gender	–0.18	0.14	–0.10	0
**Block 2**				
Time Spent on YouTube	0.15	0.06	0.20^∗^	0.07^∗^
**Block 3**				
Perceived Similarity	0.07	0.02	0.38^∗∗∗^	0.13^∗∗∗^

*All coefficients are from the full model. ^∗^*p* < 0.05, ^∗∗∗^*p* < 0.001.*

**FIGURE 1:**
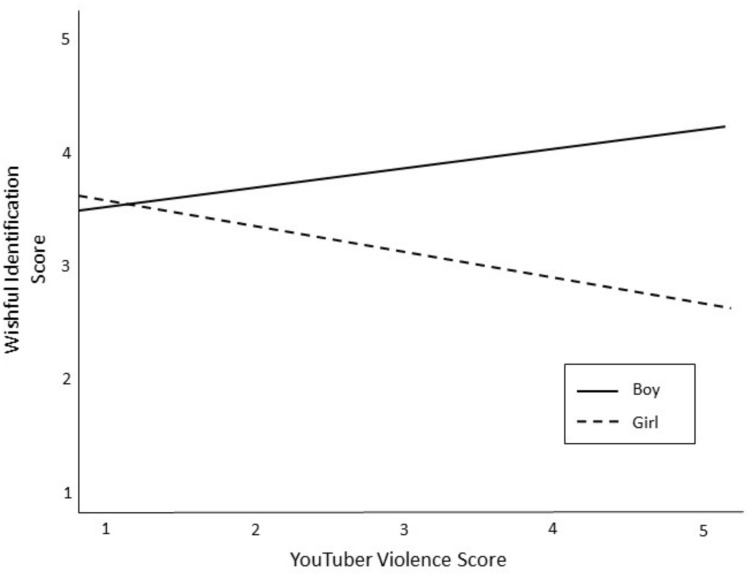
The relationship between YouTuber violence and WI as moderated by gender of child.

The third set of research questions asked about the character attributes that were related to tween boys’ and tween girls’ WI with their favorite YouTuber. After variables were mean-centered, a hierarchical linear regression was conducted to assess for main effects of YouTuber characteristics on WI and to test if child gender moderated any of these effects (see Table 4). The model controlled for child demographics (Block 1), time spent on YouTube (Block 2), and the effects of perceived similarity (Block 3). YouTuber characteristics were entered into Block 4 wherein tweens’ WI was positively predicted by YouTubers who were both funny and smart. In other words, tweens had stronger WI with YouTubers they considered humorous (β = 0.18; *p* < 0.05) and intelligent (β = 0.21, *p* < 0.05). To test if child gender moderated any of these relationships, interaction terms were entered into the Block 5 of the model. Results show that child gender significantly moderated the relationship between YouTuber violence and WI (β = −0.23; *p* < 0.05). Specifically, boys’ WI increased as YouTubers violence increased, whereas girls’ WI decreased as YouTubers violence increased (see Figure 1).

**TABLE 4:** Summary of hierarchical regression for attributes predicting wishful identification as moderated by child gender.

	**B**	***SE***	**β**	**Δ*R*^2^**
**Block 1**				
Child Race	0.07	0.04	0.15	
Child Age	–0.09	0.09	–0.08	
Child Gender	–0.23	0.15	–0.12	0.01
**Block 2**				
Time Spent on YouTube	0	0	0.13	0.08^∗∗^
**Block 3**				
Perceived Similarity	0.05	0.02	0.27^∗∗^	0.13^∗∗∗^
**Block 4**				
Smart	0.20	0.09	0.04^∗^	
Successful	0.07	0.14	0.05	
Attractive	–0.05	0.11	–0.05	
Funny	0.25	0.12	0.18^∗^	
Violent	0.15	0.15	0.11	
Popular	0.16	0.19	0.12	0.12^∗∗^
**Block 5**				
Child Gender ^∗^ Smart	0.23	0.18	0.13	
Child Gender ^∗^ Successful	–0.06	0.28	–0.02	
Child Gender ^∗^ Attractive	–0.05	0.22	–0.02	
Child Gender ^∗^ Funny	–0.37	0.23	–0.13	
Child Gender ^∗^ Violent	–0.61	0.28	−0.23^∗^	
Child Gender ^∗^ Popular	–0.22	0.36	–0.08	0.05

*All coefficients are from the full model. Child gender was coded as 1 for male. ^∗^*p* < 0.05, ^∗∗^*p* < 0.01, ^∗∗∗^*p* < 0.001.*

### Tweens Parasocial Relationships With YouTubers

Tweens could have a PSR score ranging between 1 and 5. The mean of the PSR score was 3.83 (*SD* = 0.81), indicating a tendency for tweens to experience a fairly strong relationship with their favorite YouTuber. In fact, a majority (81.6%) of the sample scored above a 3, and over half (53.1%) of the sample scored over a 4 on the scale, indicating many of the participants had strong PSRs with their favorite YouTuber.

The fourth hypothesis predicted that PSRs among tweens would be positively related to time spent on YouTube. A hierarchical linear regression was conducted to test this hypothesis (see Table 5). The first block of the analysis controlled for child demographics including age, sex, and race. None of these variables predicted PSRs. Block two included tweens’ time spent on YouTube, which positively predicted PSRs (β = 0.37, *p* < 0.001). Thus, hypothesis 4 was supported.

**TABLE 5:** Summary of hierarchical regression for time spent on YouTube predicting parasocial relationships.

	**B**	***SE***	**β**	**Δ*R*^2^**
**Block 1**				
Race	0	0.03	0	
Age	–0.09	0.08	–0.09	
Gender	–0.02	0.13	–0.02	0.01
**Block 2**				
Time Spent on YouTube	0.25	0.05	0.37	0.14^∗∗∗^

*All coefficients are from the full model. ^∗∗∗^*p* < 0.001.*

Beyond time spent on YouTube, the similarity between the tween and the YouTuber may play a role in a PSR. A hierarchical linear regression was conducted to confirm this fifth hypothesis. The first and second blocks controlled for child demographics and time spent with YouTube, respectively. The third block, perceived similarity, positively predicted PSRs (β = 0.29, *p* < 0.01) (see Table 6). Thus, hypothesis 5 was supported.

**TABLE 6:** Summary of hierarchical regression for perceived similarity predicting parasocial relationships.

	**B**	***SE***	**β**	**Δ*R*^2^**
**Block 1**				
Race	0.03	0.03	0.09	
Age	–0.12	0.08	–0.12	
Gender	–0.08	0.13	–0.05	0.01
**Block 2**				
Time Spent on YouTube	0.22	0.05	0.33^∗∗∗^	0.14^∗∗∗^
**Block 3**				
Perceived Similarity	0.05	0.01	0.29^∗∗^	0.07^∗∗^

*All coefficients are from the full model. ^∗∗^*p* < 0.01, ^∗∗∗^*p* < 0.001*

**FIGURE 2:**
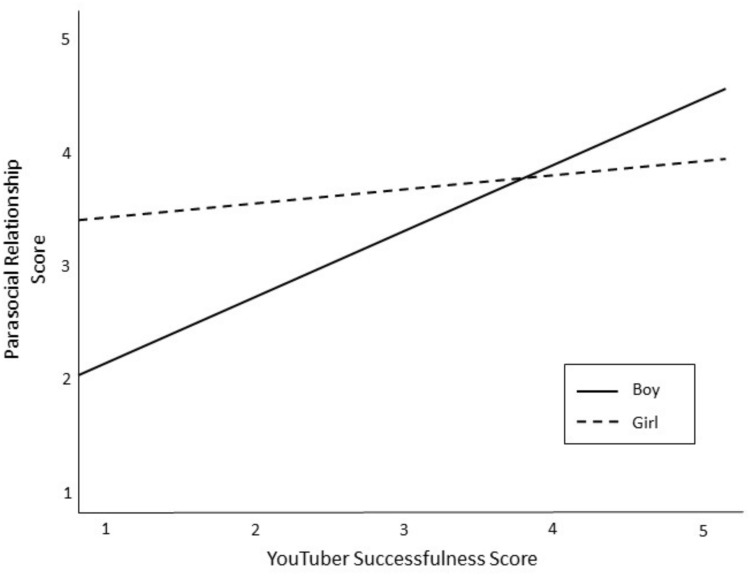
The relationship between YouTuber successfulness and PSR as moderated by gender of child.

**FIGURE 3:**
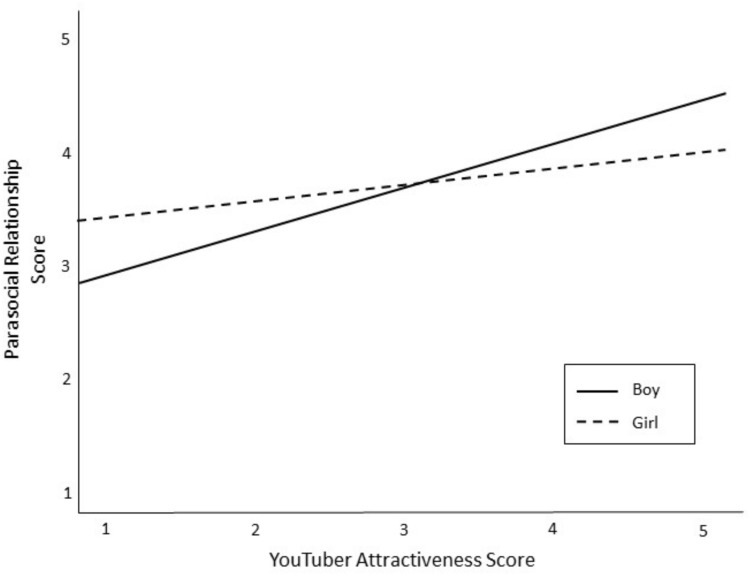
The relationship between YouTuber attractiveness and PSR as moderated by gender of child.

**FIGURE 4:**
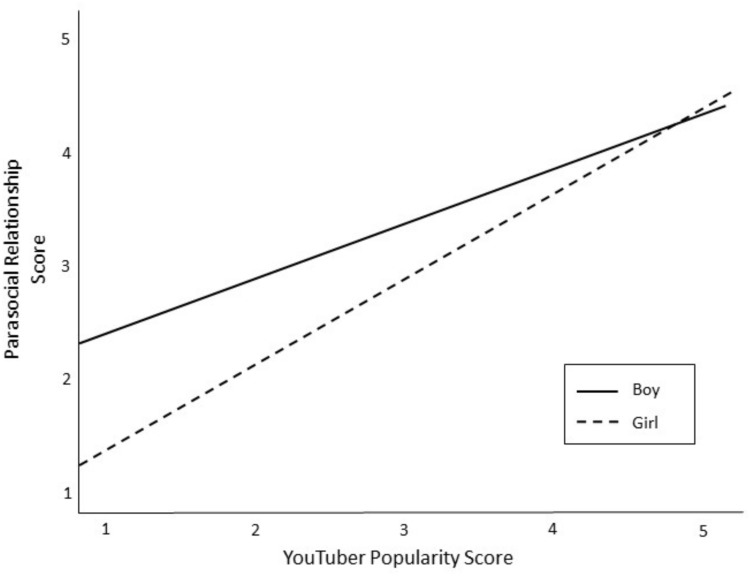
The relationship between YouTuber popularity and PSR as moderated by gender of child.

The fourth set of research questions asked which YouTuber attributes were related to tween boys’ and girls’ PSRs. After mean-centering the variables, a hierarchical linear regression was conducted to test for main effects of YouTuber characteristics on PSR and to test if gender moderated any of these effects (see Table 7). The analysis controlled for the role of demographics (Block 1), time spent on YouTube (Block 2), and perceived similarity (Block 3). There was a significant main effect for funny YouTubers, such that tweens had stronger PSR with YouTubers they considered humorous (β = 0.22, *p* < 0.01). Interaction terms were entered into the final block of the analysis. Results show that child gender significantly moderated the relationship between YouTuber successfulness and PSR (β = −0.20; *p* < 0.05). Specifically, boys’ PSR increased for more successful YouTubers (see Figure 2). Gender also approached significance as a moderator of the relationship between YouTuber attractiveness and PSR (β = −0.18; *p* = 0.05), and between YouTuber popularity and PSR (β = 0.22; *p* = 0.07). Tween boys tended to have stronger PSR for attractive YouTubers (see Figure 3), and tween girls tended to have stronger PSR with popular YouTubers (see Figure 4).

**TABLE 7:** Summary of hierarchical regression for attributes predicting parasocial relationships as moderated by child gender.

	**B**	***SE***	**β**	**Δ*R*^2^**
**Block 1**				
Child Race	0.04	0.03	0.10	
Child Age	–0.14	0.07	−0.13^+^	
Child Gender	–0.16	0.12	–0.10	0.01
**Block 2**				
Time Spent on YouTube	0.01	0	0.22^∗∗^	0.15^∗∗∗^
**Block 3**				
Perceived Similarity	0.03	0.01	0.19^∗^	0.08^∗∗^
**Block 4**				
Smart	–0.13	0.07	–0.02	
Successful	0.06	0.11	0.05	
Attractive	0.04	0.09	0.04	
Funny	0.27	0.09	0.22^∗∗^	
Violent	0.09	0.12	0.08	
Popular	0.39	0.15	0.32^∗^	0.19^∗∗∗^
**Block 5**				
Child Gender ^∗^ Smart	0.12	0.14	0.08	
Child Gender ^∗^ Successful	–0.51	0.22	−0.20^∗^	
Child Gender ^∗^ Attractive	–0.35	0.18	−0.18^+^	
Child Gender ^∗^ Funny	–0.25	0.18	–0.10	
Child Gender ^∗^ Violent	–0.02	0.22	–0.01	
Child Gender ^∗^ Popular	0.53	0.29	0.22^+^	0.07^∗∗^

*All coefficients are from the full model. Child gender was coded as 1 for male. ^+^*p* < 0.10, ^∗^*p* < 0.05, ^∗∗^*p* < 0.01, ^∗∗∗^*p* < 0.001.*

Tweens can access their favorite YouTuber across many social media platforms. Indeed, many (41.5%) of the tweens reported following their favorite YouTuber on at least one other social media platform. The most popular social media platform used to follow a YouTuber was Instagram (31.3%), followed by SnapChat (20.4%), Musical.ly (12.2%), Twitter (10.2%), and Facebook (1.4%). Research question 5 asked if following a YouTuber across other social media platforms is related to PSRs. Another hierarchical linear regression was conducted, which controlled for the child demographics and time spent on YouTube. The third block of the analysis included the total number of social media platforms used by the participant to follow the YouTuber. This variable was not significant. Thus, to answer RQ5, it appears that following YouTubers across different social media platforms does not strengthen tweens’ PSRs.

Compared to merely seeing a YouTuber across multiple social media platforms, a more powerful predictor of PSRs may be whether a YouTuber directly communicates with a tween through messages or comments. One-third (32.7%) of the participants reported leaving at least one comment for a YouTuber during the “average week.” And although it may seem like a rare occurrence, several participants (11.6%) reported that a YouTuber had responded to their comments. Sending a private message to a YouTuber during an “average week” was rarer, with only 6.8% of the tweens reporting doing so. Only 5.5% of the tweens reported receiving responses to their private messages. Hypothesis 6 predicted that receiving direct communication from a YouTuber would be positively related to tweens PSRs. A hierarchical linear regression (see Table 8) tested this hypothesis and found that after controlling for demographics, time spent on YouTube, and perceived similarity, receiving replies to private messages predicted PSRs (β = 0.18, *p* < 0.05) such that receiving more YouTuber replies to private messages strengthened PSRs. However, receiving a reply from a YouTuber to a comment made was not related to tweens’ PSRs. Thus hypothesis 5 was supported with one measure of communication (receiving private messages) but not the other (receiving comments).

**TABLE 8:** Summary of hierarchical regression for interpersonal interaction with YouTuber predicting parasocial relationships.

	**B**	***SE***	**β**	**Δ*R*^2^**
**Block 1**				
Race	0.05	0.03	0.10	
Age	–0.07	0.09	–0.06	
Gender	–0.18	0.14	–0.10	0
**Block 2**				
Time Spent on YouTube	0.15	0.06	0.20^∗^	0.07^∗^
**Block 3**				
Perceived Similarity	0.07	0.02	0.38^∗∗∗^	0.13^∗∗∗^
**Block 4**				
Responses from YouTuber on Comments	–0.04	0.07	–0.06	
Responses from YouTuber in Private Messages	0.17	0.09	0.18^∗^	0.02

*All coefficients are from the full model. ^∗^*p* < 0.05, ^∗∗∗^*p* < 0.001.*

## Discussion

The results from the current study suggest that YouTube is embedded in the lives of tweens. Most of the tweens in this sample reported watching YouTube regularly – between 30 and 60 min on the weekends and slightly less than 30 min on weekdays. The amount of time tweens spend on YouTube is interesting, but it may be equally important to know with whom they are watching. The current findings signal that co-viewing YouTube with a parent is rare, while watching alone or with friends is more prevalent. Tweens reported frequently watching YouTube on a personal device such as a mobile phone or tablet. The size and portability of those devices may decrease opportunities for parental co-viewing. If tweens are watching without adults, they may be exposed to more inappropriate content than parents realize.

Not only are tweens enthusiastic about watching YouTube content, they are also enthusiastic about their favorite YouTubers. Nearly all participants identified a favorite YouTuber, but there was very little overlap in the YouTubers who were named as favorites. Almost 100 unique YouTube personalities were listed. Many of the YouTubers, like JoJo Siwa and DanTDM appear to be developmentally appropriate for tweens. Yet, many of the YouTubers produce content for mature audiences, such as Logan Paul, who was recently punished by YouTube for posting a video featuring a dead body (Dwyer, 2018). The current study did not analyze YouTube content. Future research needs to investigate the content produced by tweens’ favorite YouTubers, as different types of content may have different effects.

Despite not knowing much about the content made by these YouTubers, we do know some things about the YouTubers themselves. The first hypothesis predicted that boys would be more likely to list male YouTubers as their favorites, whereas girls would list female YouTubers. The results supported this hypothesis. Boys’ choice of male YouTubers is consistent with previous work on mass media characters (e.g., Hoffner, 1996). Several girls also chose a male YouTuber as their favorite, which mirrors past research. However, the results of this study are the first to show that girls were significantly more likely to choose a female media personality as their favorite over a male media personality. It appears that YouTube may offer more opportunities for young girls to seek out content featuring other females.

Results from the second research question also signal gender differences. Tweens perceived male and female YouTubers to have different attributes. Female YouTubers were rated as more attractive and popular than male YouTubers, and male YouTubers were rated as more violent than female YouTubers. These results reflect a consistent pattern of gender differences between male and female characters seen across television and film in which male characters are portrayed as violent female characters are often young and attractive (Smith and Granados, 2009). Gender schema theory predicts that tweens would see differences between male and female YouTubers. These differences were aligned with stereotypes reflected in mass media outlets. Tweens could be seeking content that matches previously instilled gender stereotypes, or they may be interpreting small differences according gender stereotypes. Either way, such stereotypes on YouTube can limit tweens’ understanding of gender roles (Leaper, 2015).

### Wishful Identification

The next set of research questions and hypotheses were about wishful identification – the process of wanting to be like a media personality. In this study, tweens’ WI with their favorite YouTuber was positively related to time spent on YouTube, replicating recent findings that children who spend more time watching their favorite vlogger will feel more bonded to him or her (Folkvord et al., 2019). Tweens’ WI was also related to perceived similarity between the tween and YouTuber, which parallels the findings of other studies done on WI patterns with mass media characters (e.g., Hoffner, 1996; Hoffner and Buchanan, 2005). It appears that similar processes promote the development of tweens’ WI with mass media characters and YouTubers.

YouTuber characteristics also predicted tweens’ WI. Specifically, tweens’ identified more with YouTubers who were perceived as smart and funny. It is encouraging that tweens want to be like media personalities who showcase these positive characteristics. Outside of these shared effects, there were gender differences for WI patterns. It was anticipated that girls would identify more with physically attractive YouTubers, but this was not found. In other words, the current results do not replicate previous work wherein physical attractiveness was the sole predictor of girls’ WI (Hoffner, 1996). This change is encouraging, as prior research has shown a negative relationship between perceived physical attractiveness of media characters and girls’ body image (Levine and Harrison, 2009). Boys’ WI was positively related to YouTubers’ violence. These troubling results suggest that tween boys wish to be like characters who are violent, as well as smart and humorous. This is in contrast to previous findings, which reported character intelligence as the only predictor of boys’ WI with TV characters (Hoffner, 1996).

There is a long history of theory (e.g., Bandura, 2001) and research on how identification with violent models can lead to both short-term aggression (e.g., Funk et al., 2004) and long-term aggression (e.g., Huesmann et al., 2003). One particularly relevant study found that WI with a new media personality increased aggressive behaviors specifically among young adolescent boys (Konijn et al., 2007). The results of the current study suggest a potential for the same patterns to occur with YouTube, as boys wanted to be like the YouTubers who were funny and violent. This combination is particularly dubious, as comedy can trivialize violence and further increase the likelihood of imitation (Potter and Warren, 2006). Although these results may be cause for concern, we do not know the way violence is being portrayed in these YouTube videos, or the meaning that young viewers are making of this type of mediated violence. Future research needs to address the nature of the violence in YouTube videos popular with young audiences, as well as the effects of viewing said violence.

### Parasocial Relationships

Not only did tweens want to be like their favorite YouTuber, they also reported experiencing feelings of friendship. Results indicated that tweens are forming fairly strong PSRs with their favorite YouTuber. Much like WI, PSRs were positively related to time spent on YouTube and to perceived similarity between the tween and YouTuber. Again, these findings replicate past research in this arena and extend them into the digital realm of YouTube.

Tweens’ PSRs were predicted by YouTubers who were humorous. This finding makes sense, as research shows that people who are funny are more socially attractive and likeable (Wanzer et al., 1996). Beyond humor, tween boys and girls experienced unique predictors of PSR. Boys’ PSR was stronger for successful YouTubers, those who achieved goals, and PSR tended to get stronger for more attractive YouTubers. Meanwhile, girls’ PSR tended to strengthen for popular YouTubers, who were well-liked and respected.

When examined through the lens of gender schema theory, the results of this study suggest potential for contrasting gender socialization patterns. Not only were there stereotypical gender differences in the perceived attributes of male and female YouTubers, but tween boys and girls formed attachments based on different criteria. Gender schema theory predicts that boys will be paying close attention to the male YouTubers, learning what it means to think and behave like a male. Once the gendered schema for male is in place, this robust cognitive heuristic will be used to (a) filter future incoming information about masculine identities and (b) inform tween boys how to think, feel, and behave as a male should. Similarly, girls will be looking to female YouTubers to demonstrate important components of the female identity. Research should investigate how YouTubers function as role models and influence tweens’ identity development.

Not only do young people follow their real-life friends on social media, but the results of this study show that many tweens also follow their favorite YouTuber “friends” across social media platforms other than YouTube. We anticipated that keeping up with favorite YouTubers across social media platforms would predict tween’s PSRs, but this was not the case. The social media variable was initially computed as a sum score, giving little insight about each individual platform. Consequently, multiple *post hoc* regression analyses were conducted parsing out each social media site as a unique independent variable. None of these analyses were significant, confirming that following a YouTuber on other social media platforms is not related to PSRs.

Although following a YouTuber across platforms is not related to PSRs, it makes sense that receiving personal messages from one’s favorite YouTuber may be. Hypothesis 6 predicted that receiving communication from a YouTuber would increase the strength of tweens’ PSRs. This was partially supported. Although receiving a comment from a YouTuber did not relate to PSRs, receiving a private message from a YouTuber was positively related to tweens’ PSRs. It is noteworthy that only eight tweens reported receiving a private message from their favorite YouTuber, demonstrating how powerful this form of communication can be.

Contact between a YouTuber and a viewer pushes the boundaries of PSRs into the interpersonal realm. That is to say, what started as a one-sided relationship becomes two-sided when the YouTuber can communicate with the fan. We asked participants if they felt like their favorite YouTuber has become their friend. Over one-quarter (27.8%) of the sample replied “yes.” Many tweens may be experiencing a hyperpersonal parasocial relationship that is mostly one-sided but occasionally becomes interpersonal. Research should explore these new, hyperpersonal parasocial relationships, as they could mediate or moderate other media effects.

### Limitations

Like any study, the current research has limitations. The sample consists of tweens from a large, metropolitan area in the southern United States. It is possible that children from other locations experience attachments to YouTubers in a different way. There may also be systematic differences in which tweens opted to participate. Moreover, causal claims cannot be made from cross-sectional data and, as with all self-report research, there could be instances where mood, social-desirability bias, or other factors influenced participants’ survey responses. Longitudinal and/or experimental methods would improve future research in this area. Additionally, some measures in the study (e.g., interpersonal engagement, social media following, social viewing) are not-standardized instruments, and this is may limit the generalizability of the results. Finally, the current study operationalized gender in a binary fashion. Tweens are capable of identifying their gender as non-binary or transgender (Olson-Kennedy et al., 2016). Future research must address how gender non-conforming youth experience attachments with media personalities.

Regardless of these limitations, this study represents an initial attempt to assess tweens’ emotional connections to YouTube personalities. This gap in the literature is regrettable given that children may experience strong attachments to these Internet celebrities. Not only can tweens watch countless hours of content produced by their favorite YouTuber, but they can also buy merchandise, attend events populated with YouTubers, and tune in to see their favorite YouTubers on awards shows. As scholars learn more about PSR and WI, we are beginning to understand the power of these attachments. Consequently, YouTubers stand to act as potent role models, shaping the development of young people’s attitudes and conceptions about gender identity and what it means to be a male and female in today’s society.

## Data Availability Statement

The datasets generated for this study are available on request to the corresponding author.

## Ethics Statement

The studies involving human participants were reviewed and approved by The University of Texas at Dallas. Written informed consent to participate in this study was provided by the participants’ legal guardian/next of kin.

## Author Contributions

AT and KD were involved in the conceptualization, planning, data collection and analysis, and writing of the manuscript.

## Conflict of Interest

The authors declare that the research was conducted in the absence of any commercial or financial relationships that could be construed as a potential conflict of interest.

## APPENDIX A

**Table:** Summary of participants’ favorite YouTubers (*N* = 148).

**YouTuber Name**	**Number of Times Identified**	**Percent %**
DanTDM	6	3.7
Ali-A	5	3.1
The Odd 1s Out	5	3.1
Annie LeBlanc	4	2.5
Muselk	4	2.5
Myth	4	2.5
Ninja	4	2.5
Wengie	4	2.5
LDShadowlady	3	1.9
Rebecca Zamolo	3	1.9
Rosanna Pansino	3	1.9
Alex Wassabi	2	1.2
Alisha Marie	2	1.2
Cole and Sav	2	1.2
Dakotaz - BCC Trolli	2	1.2
F2 Freestylers	2	1.2
Jacksepticeye	2	1.2
Jake Paul	2	1.2
Julia (Crafty Girls)	2	1.2
Liza Koshy	2	1.2
Logan Paul	2	1.2
MatPat (Game Theory)	2	1.2
Natoo	2	1.2
PewDiePie	2	1.2
Preston Playz	2	1.2
Shane Dawson	2	1.2
Sofie Dossi	2	1.2
Squeezie	2	1.2
5minute crafts	1	0.6
AlexACE	1	0.6
Alexis and Teddie	1	0.6
Aphmau	1	0.6
As Is (Freddie)	1	0.6
Beasty Games	1	0.6
Binging with Babish	1	0.6
Blitz	1	0.6
Camodo Gaming	1	0.6
Carina Garcia	1	0.6
Ceeday	1	0.6
CGP Grey	1	0.6
Chris Stuckmann	1	0.6
Clifford Owusu	1	0.6
Collin Keyes	1	0.6
Coyote Peterson	1	0.6
Crafty Girls	1	0.6
Dangmattsmith	1	0.6
Draegast	1	0.6
Dude Perfect	1	0.6
Emma Chamberlain	1	0.6
Evantube	1	0.6
Fitz	1	0.6
Funnel Vision	1	0.6
Hunter Haynes	1	0.6
Infinite Lists	1	0.6
Jackie Aina	1	0.6
Jelly	1	0.6
Jojo Siwa	1	0.6
Kayla Davis	1	0.6
Kids React	1	0.6
Kitty Plays	1	0.6
Lachlan	1	0.6
Lele Pons	1	0.6
Madden Mobile Goods	1	0.6
Markiplier	1	0.6
Mathias	1	0.6
Matt Meese	1	0.6
Meredith Foster	1	0.6
Moose	1	0.6
Nathan/Unspeakable	1	0.6
Nicole Skyes	1	0.6
Onyx Kids/Shiloh	1	0.6
Pixelated Apollo	1	0.6
Popular MMOs (Pat)	1	0.6
Roland Bauduin	1	0.6
Royal Opera House	1	0.6
Ryan Higa	1	0.6
SaraBeautyCounter	1	0.6
Simplynailogical – C	1	0.6
Skits4Skittles	1	0.6
Skyaart TV	1	0.6
SSundee	1	0.6
Stampy	1	0.6
Studio C	1	0.6
Stupid Things With S	1	0.6
Tekking 101	1	0.6
The Seven Girls	1	0.6
TheGamingBeaver	1	0.6
Thinknoodles	1	0.6
Today I Found Out	1	0.6
Trend Crave	1	0.6
Unlisted Leaf	1	0.6
Vennessa Merrell	1	0.6
Yannick Bergeron	1	0.6
Yassy	1	0.6
Yolanda (Cake Maker)	1	0.6
Zach Hample	1	0.6
Zach King	1	0.6
